# How digital medicine redefines the landscape of care: Ethical and legal consequences in the case of psychiatry

**DOI:** 10.1177/20552076261478845

**Published:** 2026-08-12

**Authors:** Sandra Anna Just, Brita Elvevåg

**Affiliations:** 1 Department of Clinical Medicine, 8016 UiT The Arctic University of Norway, Tromsø, Norway; 2 Department of Psychiatry and Neurosciences, Charité Campus Mitte, 14903 Charité – Universitätsmedizin Berlin, Berlin, Germany

**Keywords:** care, digital medicine, ethics, artificial intelligence, psychiatry, chatbots, law, phenomenology

## Abstract

Care is central to medical practice. However, care is currently being re-defined due to the computational logic that is central to digital medicine. For those with serious mental illness in psychiatry, the new landscape of care has significant ethical, legal, and phenomenological implications. The obvious and less obvious differences in care between human caregivers and technology warrant reflection. This commentary examines the nature of care in digital medicine through a case study of voice-based chatbots used in the context of mental health. Several recommendations are derived to reconcile human-centered care with risks in digital medicine.

This commentary offers a conceptual contribution to a rarely examined issue, namely how the logic of digital medicine reshapes the very concept of medical care, and what ethical, legal, and phenomenological consequences follow. We use voice-based chatbots in psychiatry as an illustrative case, drawing on the literature to develop the argument. Our aim is to offer one perspective that we consider to be useful for thinking about the future of care in digital medicine.

## 1. What is care?

The concept of care is intuitive yet opaque. Drawing on the care literature (for an overview^1^), two broad perspectives can be distinguished. First, care as it is experienced by the care recipient: feeling ‘taken care of’ is central in human experiences, from early in life – an infant’s survival depends on it. This perspective, the patient’s lived, embodied experience, is central to the phenomenological tradition in medicine.^2^ The second perspective concerns care as it is enacted by a caregiver: ‘caring for others’ describes regard, benevolent intention, and commitment toward another human being. This is in line with the medical ethics literature, which – albeit diverse – shares an understanding of caring as relational and motivated by a commitment to another person. The health *care* system institutionalizes care and differentiates between those delivering (nurses, doctors, therapists) and those receiving care (patients). Receiving care entails vulnerability requiring legal and ethical safeguards (e.g., confidentiality, consent, and respect for autonomy). Phenomenological medical studies emphasize that ‘caring for others’ is a multimodal experience, involving speech, sight, and touch.^3^ Patients report feeling ‘taken care of’ when recognized as a human who is taken seriously and attended to.^4^ Psychiatric patients similarly describe ‘good care’ as a dignified experience of respect, safety, and validation.^5^ Clearly ‘treatment’ is neither necessary nor sufficient for the experience of care. While an effective treatment can evoke the experience of care, a patient may feel well taken care of without a prescription. Similarly, an established therapeutic relationship between caregiver and those receiving care can contribute to feeling taken care of but is not necessary. Patients can feel taken care of by strangers if treated competently and with respect.^4^ Thus, beyond treatment plans and relationships, the concept of care in medicine is a commitment to recognizing and responding to another human’s vulnerability (see Glossary in Table 1).

**Table 1.** Glossary of terms used in this article.

Term	Definition
Care	*The enacted aspect of care refers to the commitment of recognizing and responding to another person’s vulnerability: a care-giver shows concern and regard for a care-receiver, and performs concrete acts to sustain or improve their well-being. Care in medicine goes beyond treatment – a person may be effectively treated without feeling cared for, and may feel cared for without treatment. This felt sense of being ‘taken care of’ is the experienced aspect of care.*
Therapeutic relationship	*The relationship between health care professionals and their patients can nurture the feeling of being taken care of. Traditionally understood through a paternalistic model (where the doctor ‘knew best’), it is now recognized that medical practice can be more effective when these relationships are built on mutual trust. In mental health, the therapeutic relationship refers to the alliance between a therapist and their client within a psychotherapeutic setting. This relationship is characterized by empathy, positive regard and shared goals as well as legal and ethical safeguards, such as confidentiality, informed consent, and respect for autonomy. A therapeutic relationship should be distinguished from relationality: the former is an established, specific relationship, whereas the latter is the commitment to another human being inherent in any act of care. Care therefore requires relationality but not necessarily an established therapeutic relationship.*
Treatment	*Usually refers to an intervention – digital, pharmacological, social, therapeutic, surgical – that is delivered by healthcare professionals with the intention to treat a patient with a clinically relevant condition. In the context of this comment, it is important to differentiate a digital tool that ‘fixes’ a putative problem versus a tool that acts as the vehicle to support or deliver the treatment (e.g., a telephone to have a supportive conversation, or to connect the person needing treatment to the professionals or service that can provide it). For example, voice-based chatbots (the case study in this comment) may ‘fix’ mental health problems of users through their generative conversational capabilities*.
Effectiveness vs. efficiency	*Effectiveness quantifies the extent to which a treatment achieves its intended outcomes, such as reducing symptoms or improving quality of life – regardless of the resources required to obtain those results. In contrast, efficiency quantifies those resources, assessing the ‘value’ of treatments in terms of time, effort, or money needed to achieve a treatment goal*.
Bias	*Medical care is biased when it is influenced by rules, principles or information (e.g., stereotypes) that are inappropriate in a given situation and influence decision-making processes in potentially harmful ways. Biases are often implicit and can arise from the person delivering care (e.g., a clinician) or from the way decision-making was designed. A data-driven system or tool will be biased if its training data contained prejudiced, distorted or simply wrong information or has not been updated when the knowledge base changes. Discrimination occurs when individuals or groups are treated differently and to their disadvantage on the basis of biases about protected characteristics such as age, gender, disability, ethnicity, religion, or sexual orientation.*

## 2. How does digital medicine define care?

Digital medicine merges empiricism of evidence-based medicine with the logic of computer science, whose core method is data-driven detection and problem-solving. Even approaches designed to preserve clinical uncertainty and individual variability (e.g., human oversight or longitudinal symptom monitoring) operate within this computational logic: variability and uncertainty are understood as parameters of a problem to be modelled and solved, rather than as defining aspects of caring for humans. Unlike other medical fields, digital medicine therefore usually focuses on tools, designed for ‘solving a problem’ (i.e. an illness). This goal is achieved by applying such tools in diagnostics, prevention and treatment, after evaluation in clinical trials. Digital medicine aims to deliver ‘new systems of care’ tailored to the individual, efficiently, proactively and economically.^6^ Crucially, it moves beyond digitizing existing workflows. Digital medicine reshapes how medical tasks are performed^7^ as the tools gather and analyze data, generate insights, and often incorporate artificial intelligence (AI) to support or automate clinical decision-making, pattern detection and outcome prediction.^8^ While many argue that such tools should only support, not replace clinicians,^9^ some tools, such as fully automated therapeutic programs, are designed to work without a human clinician.^10^

Digital tools in clinical use today do not perform diagnostics, prevention, or treatments but narrower tasks that support, not replace, human clinicians – notably pattern detection in medical data (e.g., imaging).^11^ These tools have been implemented where problems can be clearly defined, quantified and measured, such as oncology, ophthalmology, and pharmacology – and less so in other fields notably in psychiatry. It should be noted that there are numerous scientific studies that have developed and/or evaluated more complex digital tools in psychiatry, such as clinical decision support systems.^12–14^ However, hardly any of these tools have been implemented in clinical settings and those that have, again, focus on clearly defined problems and solutions (e.g., medication management).^15,16^

This focus on problem-solving reveals a limitation. For the most part, digital medicine does not focus on care – and thereby the emerging field effectively redefines the very concept of care itself. While most digital tools are not intentionally designed to replace relational care, they still adopt a computational logic: in order to be digitized, something must be computationalized – translated, at the most basic level, into code and numbers, which requires specifying a problem with defined parameters. These algorithms are not neutral but reflect aspects of medicine deemed relevant.^17^ The problem-solving approach treats illnesses as objectively identifiable entities. When developing a tool that targets an illness, care can no longer be viewed as a relational concept – as it is the illness that is treated, not the human. This is an ideal foundation to develop a commercial product for a potential consumer where the goals are efficiency and monetization.^18^ While medical care has always had to operate under economic pressures, these are amplified under the numerical logic underpinning digital medicine – its ‘profit-driven pressures’^19^ – at the expense of care. These shifts are not merely theoretical but become embedded in laws and regulations, determining what care is possible. For example, while regulation of medical AI remains fragmented in the United States, Europe’s AI Act requires complex regulatory processes for medical devices.^20^ However, a growing number of legal scholars argue that the AI Act’s approach to establishing risk of the technology (including medical applications) favors private entities over patient autonomy. The AI Act defines requirements for developers of AI systems in healthcare but does not address the vulnerable position of patients themselves.^21^ This impacts the dynamics of care itself.^22^

## 3. The case of psychiatry

Globally approximately 1 billion people live with mental illness, and poor mental health is projected to cost the global economy around $6 trillion per year in lost productivity and health by 2030.^23^ The case below of psychiatry exemplifies how care is affected by technology.

Psychiatry has a long history of de-humanizing and naturalistic conceptions of mental illness. Recently, the field has moved towards an integrative view that embraces human complexity.^24,25^ Unlike some areas of medicine, mental illnesses cannot be ‘detected’ from blood tests or various scans. Their ‘detection’ is complex: diagnostic boundaries remain fluid and contested.^26^ And the individual’s experience of mental illness is shaped by personal and cultural meaning as well as power imbalances in the societal context.^25^ Mental illness is not easily translated into a discrete, solvable problem.

This understanding of complexity is threatened by an increased use of computational logic within psychiatry. This is because the process re-introduces simplistic conceptions of detectable and solvable problems that by default define mental illness. When digital medicine follows this computational logic and treats heterogenous conditions such as schizophrenia or depression as clearly defined ‘problems’, complexity is lost. In schizophrenia for example, recovery is an intricate, individual journey, involving among others the person’s self-image, relationships, and societal participation. This journey cannot be computationalized, but relapse can: it is a discrete, measurable event with defined parameters. Digital tools can be developed for this clearly defined problem, offering a ‘solution’. This reveals a mismatch between complex conditions and simplified solutions. These solutions however can be packaged straightforwardly into research programs. They are easier to fund and capitalize on, while more complex forms of support for people with mental illness are not prioritized. Defining the ‘problem’ therefore has far-reaching consequences for people living with mental illness and for the type and level of care they experience.

The resulting redefinition of care in digital psychiatry is both exciting and alarming. Undeniably digital medicine offers numerous exciting opportunities for improving care.^27^ However, implementing digital tools in mental health without examining how they redefine care is both unfair to patients and potentially dangerous. Put bluntly, human dignity is at risk if care, as a fundamental aspect of the human experience, is ignored in favor of data-driven, often economically motivated, logic.^18^ This shift can produce ‘opaque institutions’, governed by algorithms and economic logic that are difficult to understand, especially for vulnerable patients who depend on medical care and ‘are already at severe epistemic disadvantage’.^28^ For patients, the subjective experience of dealing with an opaque system consists of being monitored, evaluated, categorized or advised without knowing that an algorithm was involved, how it came to a decision or how to get information on it. This opacity reinforces existing power imbalances in health care systems and can dehumanize the very people it is meant to support.

### 3.1. Case study: Mental health conversations with voice-based chatbots

Chatbots with synthetic voices that can be used to discuss mental health issues exist on a broad spectrum with different levels of sophistication and regulation. At one end are highly specialized chatbots designed for delivering therapy and if certified can be marketed as medical devices. At the other end of the spectrum are unregulated informational tools and general-purpose AI-based chatbots (e.g., ChatGPT). Although these unregulated systems are not intended for clinical use, individuals nonetheless discuss mental health issues with them.^29,30^ Examining these chatbots showcases how technology reshapes care and the cascade of phenomenological, ethical and legal consequences. While some observations below (e.g., privacy of speech data) are specific to voice-based chatbots, most of the examined issues extend beyond them: ethical, safety and equity concerns apply across digital medicine, and simulated empathy and the resulting need for technology disclosure apply to any tool presenting an interactive interface.

A central question concerns the phenomenology of talking with a machine. We argue that human therapists can care for others, but machines cannot. AI systems are ‘irrelational’,^31^ incapable of caring in the relational, embodied sense developed here. Yet voice-based chatbots can convincingly simulate some conditions of care: they excel at simulating empathy by personalizing responses and maintaining consistent ‘attention’. Humans tend to project emotions onto inanimate objects and can become emotionally attached to a chatbot.^32^ Some individuals who struggle with human interaction may even prefer and benefit from interacting with a chatbot.^33^ Taking the user’s lived experience seriously, this relational engagement is phenomenologically real and functionally meaningful for them, regardless of whether the system feels anything.^34^ The experience is nonetheless asymmetric: it is experienced as relational on the user’s side, yet unreciprocated as the machine cannot care. Although human-machine relationships are fundamentally different from human-human relationships,^35^ the phenomenological experience of this relationship may thus still be one of ‘care’ – a real lived experience, but exclusively on the side of (vulnerable) human being.

This simulation of putative care introduces significant ethical challenges, compounded by the absence of regulatory oversight and clear accountability for most chatbots.^36^ Users may feel cared for without the protections, professional standards, or ethical obligations that govern human therapeutic relationships (e.g., confidentiality, duty to warn). When a person is harmed through talking with a chatbot about their mental health, it remains an open question who is accountable.^19,37^ Moreover, without explicit ‘bot disclosure’, (vulnerable) users may not realize they are interacting with AI – a ‘counterfeiting of humanity’ that is unfair to them und undermines their ability to make informed choices.^17^ A misperception in digital medicine that this ‘interaction’, albeit significantly cheaper than human care, is an acceptable proxy will further reduce the value of care.

Relationships with machines carry distinct risks that differ from human therapeutic relationships.^35^ Ensuring data privacy is more complicated for voice-based than text-only chatbots^38^ as speech contains identifiable information^39^ on someone’s age, gender, language background, emotions, health status, and other personal characteristics. AI-based chatbots introduce additional risks and lack systematic evaluation for mental health purposes. They can perpetuate biases embedded in training data and hallucinate, producing false or potentially harmful clinical advice.^38^ The opacity of these ‘black box’ systems makes it difficult to trace how therapeutic recommendations are generated or to prevent unhelpful or dangerous responses.^30^ Naturally, technical safeguards for chatbots are already in place and continuously developed further, including improved handling of sensitive conversations.^29,40^ Yet these safeguards solve problems that the new solutions themselves create and do not address the discussed redefinition of care.

Finally, when it is argued that digital systems of care are ‘better than nothing’,^35^ it is important to note who is excluded from them. Digital tools may expand access where mental health professionals are scarce but this does not, by itself, expand access to care – it may even obscure the gap it appears to close. Any potential increase in equity would be uneven as these systems are mostly designed in English or other majority languages^41^ and used by people with digital skills and resources. While many people may benefit from a digital therapist speaking their native language, the performance of multilingual chatbots decreases for low-resource languages and they lack cultural competency. Also, technological innovations in medicine are not designed for and often exclude the elderly and those with limited digital literacy.^42^ If tools work less well for certain groups, the inequity compounds: those with less access to human care are offered a substitute that was not built for them.

## 4. Recommendations

Digital tools cannot replace the human capacity to ‘care’. As illustrated with the case study of voice-based chatbots, this has implications on different levels: The redefinition of care and the subsequent questions of access (i.e., equity), ethics, risks, and regulation apply across digital medicine. The need to protect individuals from a ‘counterfeiting of humanity’ applies to any tool that interacts with its users. A narrower set of issues, such as speech data privacy, is specific to speech-based systems though not unique to chatbots.

### 4.1. So how should the relationship between digital medicine and care be shaped in the future?

**Figure 1.**
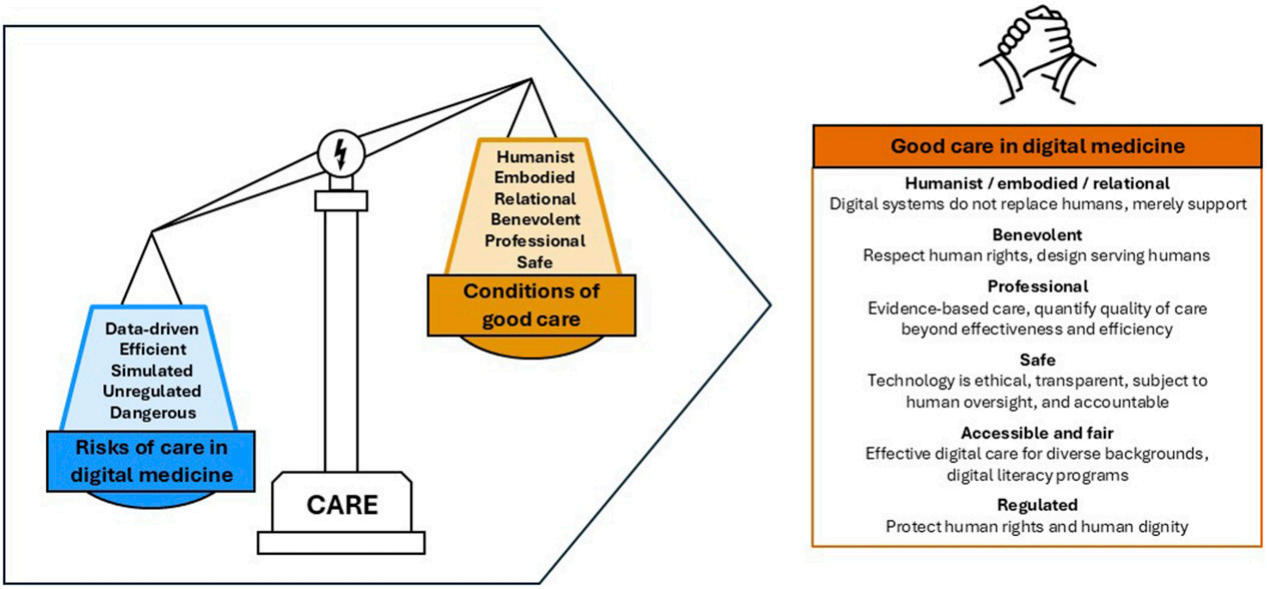
Today there is an imbalance between the challenges of digital health and the necessary conditions of care in medicine. Challenges in digital medicine are its data-driven logic that focuses on problem-solving and efficient tools. Digital tools can simulate care and humanity. They harbor problems of fairness and access and many tools are unregulated and potentially dangerous if used by vulnerable people. In contrast, the conditions of care in medicine are that it is practiced with a recognition for another human’s dignity, vulnerability, and complexity, in an embodied and relational manner, with benevolent concern as well as in an effective and competent manner within ethical and legal safeguards. The way forward for developing a concept of good care in digital medicine is to preserve what makes care humane and to integrate ethical and regulatory solutions to digital medicine’s challenges and risks.

Good science and practice in digital medicine necessitates the inclusion of domain knowledge from clinicians. In psychiatry this involves knowing the field’s history to prevent dehumanizing and simplistic conceptions of mental illness. The complexity of humans and their minds cannot be overstated and must be embraced when developing tools. Quantifiable metrics of effectiveness and efficiency remain important in evidence-based digital medicine, but so is awareness of what this may miss. Measuring the experience of care and dignity in clinical settings is needed. Inspiration can be derived from the recovery literature that moves beyond evaluating treatments solely in terms of symptom reduction. Figure 1 illustrates the different criteria for implementing good care in digital medicine, namely that care remains relational and embodied, with a benevolent intention, that it is professional and safe as well as regulated and fair with clearly defined accountabilities. Digital medical tools must be designed to support clinicians rather than eliminate human relationships or reduce clinicians to facilitators of machines. This approach supports ‘prohuman development paths’ that prioritize humanism over efficiency and profit.^31^

Regulations similarly must address not only safety and effectiveness but also human dignity. This includes establishing accountability when digital systems cause harm, ensuring transparency about AI involvement, and implementing data privacy protections. While human-delivered care is heterogeneous and clinicians can make mistakes, they are held accountable for them. Individuals must have the right to receive care from humans, especially in situations that may generate ‘powerlessness, humiliation, or anxiety’.^18^ This protection is particularly crucial in psychiatry. Subjecting people with mental illness to data-driven care compounds existing power imbalances and can be experienced as inherently humiliating.^18^ Being informed about how AI impacts their care has been suggested as a patient right. How patients are informed depends on the specific context and may vary from a mere notification to comprehensive consent procedures.^43^ The regulation of technology used for mental health support (e.g., chatbots), should not be left to private companies. The stakes are too high to allow profit interests to determine how vulnerable individuals are protected.

Within clear regulatory safeguards, individuals themselves (researchers, clinicians, people with mental illness, everyday users) must be encouraged and enabled to understand the changing care landscape. This requires investment in digital literacy that fosters understanding how systems work, their limitations, and risks. Personalized medicine implies that some individuals benefit while others do not. Individualized digital medicine should inform decisions about receiving care through technology that is fair, transparent, subject to human oversight, clearly regulated, and accountable.

## 5. Conclusion

Combining care and digital medicine is possible (see Figure 1). If the premise is accepted that care cannot be mechanized, quantified, and optimized purely for efficiency, then something fundamental about medical practice is maintained: the commitment to recognizing and responding to human vulnerability with authentic presence and regard. Only humans can provide medical care.
